# Evaluation of serum complement C4 among COVID-19 patients in Khartoum state: a case control-based study

**DOI:** 10.12688/f1000research.132670.1

**Published:** 2023-04-21

**Authors:** Mohammed Yahya, Yousif Ali Rahamtalla, Nasr Mohammed Nasr, Babbiker Mohammed Taher Gorish

**Affiliations:** 1 Department of Microbiology, Sudan University of Science and Technology, Khartoum, Khartoum, Sudan; 2 Department of Microbiology, Alzaiem Alazhari University, Khartoum, Khartoum, Sudan; 3 Department of Medical Microbiology, Omdurman Islamic University, Omdurman, Khartoum, Sudan

**Keywords:** Complement C4, Sudanese, Covid-19

## Abstract

**Background:** The complement system is made up of an abundance of unique plasma proteins that play an important role in innate immunity and inflammation, aiding in the fight against pathogenic microbes and viral diseases. The purpose of this study was to evaluate the serum complement C4 concentration in COVID-19 patients in Khartoum and compare them to healthy controls.

**Methods:** A total of 100 samples were collected, 50 samples from COVID-19 patients who presented as cases and 50 samples from people who were evidently healthy. Overall, 33 (66%) the patient populations in the case group were not in the hospital’s intensive care unit (ICU), compared to 17 (34%) who were. The concentrations of C4 in each serum sample were calculated in milligrams per deciliter. SPSS version (20) was used to analyze the data.

**Results:** The means level of complement C4 (mg/dL) were 37.44±18.618, 23.90±10.229 in the case group and in the control group, respectively. There was a statistically significant difference in complement C4 level between case and control (p-values ≤0.01). In addition, the mean complement C4 level in the ICU and non-ICU case groups was 25.00±17.85 and 43.85±15.712 mg/dL, respectively. There was a statistically significant variance in complement C4 level between ICU and non-ICU (p-values ≤0.01). Furthermore, the cases were divided into four age groups: 20-40, 40-60, 60-80, and over 80 years old. The one-way ANOVA test showed no statistically significant differences between age categories in complement C4 level (P = 0.735)

**Conclusions:** The case group had a higher mean level of complement C4 than the control group, which could be understood by the stimulation of the complement cascade during the COVID-19 illness. Furthermore, the complement C4 level in severe COVID-19 patients was lower than in non-severe COVID-19.

## Introduction

In the last twenty years, endemic cases of the Middle East respiratory syndrome (MERS)-CoV in 2012 and severe acute respiratory syndrome coronavirus (SARS-CoV) in 2003 both occurred, with case mortalities ranging from 14–15% to 35%. ^1^ At the end of December 2019, a new coronavirus was determined to be the root of a cluster of pneumonia cases with an unknown cause in Wuhan, Huanan Fresh fish Supplier Market, the city in China’s Hubei Province that served as the initial site where cases of coronavirus disease 2019 (COVID-19) were identified. ^2^ The novel coronavirus spread rapidly, causing an epidemic in China, and a pandemic with rising incidence in many other nations all over the globe. ^2^ Symptomless infection, mild upper airway disease, severe and highly contagious pneumonia, respiratory distress, and sometimes even fatality are all on the clinical phenotype of SARS-CoV-2 infection. ^3^

Initially, a link to a seafood restaurant in Wuhan that sold live animals and was frequented or worked at by the majority of the previous pneumonia patient populations was noticed. But as the pandemic disease spread, person-to-person transmission took over as the main way it was spread. Large droplets released during sneezing and coughing by symptomatic patients help spread the COVID-19 infectious disease, but transmission can also occur from people who are symptomless before developing symptoms. ^4^ The COVID-19 virus can cause clinical and pathological lung lesions that resemble those seen in other types of acute respiratory distress syndrome (ARDS) because it enters lung tissue after sticking to viral Spike proteins (S) and ACE2 receptors. ^5^

In order to eliminate invasive pathogens, complement pathways, a crucial part of the innate immune system, are activated by coronaviruses. ^6^ The main complement molecules C3 and C4 are cleaved by the proteolytic process when the complement system is activated, resulting in the cleavage products C3a, C3b, C4a, and C4b, which can cause the recruitment of inflammatory cells and the activation of neutrophils. ^7^ Patients with mild COVID-19 have shown higher levels of C3 and C4 complement, whereas those with severe COVID-19 had lower levels, which might be clarified by higher consumption from the formation of immune complexes. ^8^

In light of this, the goal of this study was to compare the serum complement C4 levels of COVID-19 patients in Khartoum to those of healthy controls. Additionally, we compared the COVID-19 patients’ levels of C4 based on the severity of their illnesses.

## Methods

### Ethics approval and consent to participate

The Ethics Committee of Sudan University of science and technology approved this research (approval number: 112BNC/12/2021; approval date: 01/12/2021). In addition, written approval was obtained from the Jabra hospital of Emergency and Trauma manager (approval number: JET 36 in December 2021). Respondents provided written informed consent after being guaranteed that only investigators would have access to the data and that it would only be used for investigation. Respondents could fill the questionnaire privately. All procedures in this study were carried out in conformity with the rules and regulations specified in the Helsinki Declaration that were applicable.

### Study setting

During the period from January to April 2022 a total of 100 subjects were involved in this study, of which 50 were cases confirmed to have COVID-19 infection and another 50 apparently healthy control were the control group. Cases were matched in sociodemographic characters. Cases and control were randomly selected from patients attending Jabra hospital of Emergency and Trauma. Gender was informed based on the self-administered questionnaire filling.

### Inclusion and exclusion criteria

The study included patients diagnosed with COVID-19 and apparently healthy controls. Patients who had received any care before being admitted other than antipyretics, had any recorded coinfections, or had immune dysfunction or malignant neoplasms were excluded.

### Sample collection and analysis using DIRUI CS-T180

A volume of 3 mL of blood was collected in plain containers from both patients and controls at Jabra Hospital of Emergency and Trauma. Then the samples were centrifuged, serum separated and stored at -80°C until analysis. C4 ELISA kit was obtained from Abcam (United Kingdom). All the protocols were done according to the manufacturer instruction. After that all reagents were brought to 37°C for warming, then 800 μL from R1 (Diluent) were added to 20 μL from the sample or calibrator and 200 μL from R2 (Antibody); finally, the absorbance of calibrator and sample had been read. The concentration of C4 was obtained by calculating the differences between calibrator and sample reading.

### Data analysis

The Statistical Package for Social Science (SPSS) Version 20 was used for data analysis. The data was presented as mean and standard deviation, frequency; Pearson’s correlation, T – test results, and one way Anova have been used as specific tests with a significant threshold value of 0.05 (P-value≤0.05).

## Results

In this study, 100 volunteers between the ages of 21 and 92 were enrolled, of whom 50 were COVID-19 patients with a mean age of 60.74 **±**18.044 years, 33 (66%) men, and 17 (34%) women. Additionally, there were 50 individuals who appeared to be in good health; their mean age was 59.68 **±**19.464 years. Of these patients, 33 (66%) were men and 17 (34%) were women ( Table 1). Regarding the case group, there were 33 (66%) non-ICU patients and 17 (34%) ICU patients ( Table 2). In the case group and control group, the mean levels of complement C4 (mg/dL) were 25.00±17.85 mg/dL and 43.85±15.712 mg/dL respectively. Between the case and control groups, there was a statistically significant difference in complement C4 level (P-value=0.00) ( Table 3).

**Table 1.** Mean ± SD of age and gender distribution among the study groups.

Study group	Mean±SD of age/years	Male	female	Total
Case group	60.74+18.044	33(66%)	17(34%)	50(100%)
Control group	59.68+19.464	33(66%)	17(34%)	50(100%)
Total		66	34	100(100%)

**Table 2.** Sex frequencies in ICU and non-ICU in case group.

Cases	Gender		Total
	Male	Female	
ICU	8 47.1%	9 52.9%	17 34%
NON-ICU	25 75.8%	8 24.2%	33 66%
Total	33 66%	17 34%	50 100%

**Table 3.** Comparison of complement C4 level between case and control.

	Group	N	Mean	Standard deviation	P-value
Complement C4 level (mg/dl)	Case	50	37.44	18.618	0.00
	Control	50	23.90	10.229	

Males and females in the case group had average complement C4 levels of 40.15 **±**20.095 and 32.18±14.466 respectively. There was no discernible gender difference in the complement C4 level among the cases group (P-value=0.153, Table 4). Based on their age, the cases were divided into four groups: the first group was made up of patients that were between the ages of 20 and 40; the second group was made up of patients between the ages of 40 and 60; the third group was made up of cases between the ages of 60 and 80; and the fourth group (80-100 years). The level of complement C4 did not significantly differ between age groups, according to a one-way ANOVA test (P-value=0.735, Table 5). The case group’s average complement C4 levels were 25.00±17.85 mg/dL and 43.85±15.712 mg/dL respectively. There was a statistically significant difference in the complement C4 level between ICU and non-ICU P-value was 0.00 ( Table 6).

**Table 4.** Comparison of complement C4 level among cases group based on their sex.

Study variable	Sex	N	Mean	Standard deviation	P-value
Complement C4 level (mg/dl)	Male	33	40.15	20.095	0.153
	Female	17	32.18	14.466	

**Table 5.** Comparison of complement C4 level among cases age groups.

Age groups	Mean	Standard deviation	P-value
20-40	33.14	19.937	0.735
40-60	41.29	18.307	
60-80	37.68	17.935	
80-100	33.29	22.470	

**Table 6.** Comparison of complement C4 level among cases group according to the severity of disease.

Study variables	Type of patient	N	Mean	Standard deviation	P-value
Complement C4 level (mg/dl)	ICU	17	25.00	17.850	0.00
	Non-ICU	33	43.85	15.712	

## Discussion

Immunoassays are frequently used in clinical practice to measure complement C4 and determine and track complement activation. A decrease in serum C4 levels brought on by increased immune system C4 consumption. Complement C4 testing in COVID-19 patients may be able to shed light on how clinical risk is balanced between normal and abnormal complement activation. ^9^ In the current study, in serum samples, complement C4 levels were assessed in 50 COVID-19 patients and 50 healthy individuals. The findings showed that there was a significant difference between the case group and control in terms of the mean complement C4 level (P-value=0.00). Zinellu and Mangoni (2021), showed there was a noticeably higher level of complement C4 in the case than in the control, which supports this finding (P-value less than 0.05). In the current study, patients with severe COVID-19 disease had lower complement C4 levels than patients with less severe COVID-19 disease, and this difference was significant (P-value=0.00). This finding concurs with that of Ghazavi and colleagues in Iran, who discovered that complement C4 levels were lower in COVID-19 patients with severe disease (P=0.014) and higher in those without severe disease (P-value 0.012). ^8^ Al-Hakeim and his colleagues also discovered in Iraq that patients who spent longer than two weeks in the hospital had lower complement C4 levels than those who were discharged earlier (P-value less than 0.05). ^10^ This result supported the findings of Al-Hakeim and his colleagues in Iraq, who found no correlation between the mean complement C4 levels across age groups and sex (P=0.681). ^10^ Males and females in the case group had average complement C4 levels of 40.15 **±**20.095 and 32.18±14.466 respectively. There was no discernible gender difference in the complement C4 level among the cases group. Prior clinical evidence has revealed that, while males and females share a comparable illness rate, males are more likely to be hospitalized, experience a more severe illness progression, and die at a greater rate than females. ^11^

A study with a large number of patients in the asymptomatic and ICU groups using COVID-19 is recommended to generate clearer and more significant results. Due to funding limitations, this research was limited to 50 COVID-19 patients. As a result, it is recommended that similar studies be conducted on a greater number of COVID-19 cases. Also, research on the other complement components would be valuable in order to acquire a better understanding of the complements pathway’s involvement. There is considerable debate over how soon complement overuse must be monitored and which components of complement consuming must be evaluated.

## Conclusions

Comparing the case group to the control, there was a higher mean level of complement C4, which can be attributed to the activation of the complement system during COVID-19 infection. Furthermore, the complement C4 level was lower in patients with severe COVID-19 particularly in comparison to those with non-severe COVID-19, which could be because the immune system of patients with severe infection consumes more complement C4.

## Authors’ contributions

YA performed main experiments, YA, NM, MY, BG collected’ samples and information. MY, designed the experiments and wrote the manuscript. All authors read and approved the final manuscript.

## Data Availability

figshare: Complement C4 in COVID 19 patients in Khartoum case-control study, https://doi.org/10.6084/m9.figshare.22249990.v1. ^12^ The project contains the following underlying data:
- Mohammed Yahya data.xlsx (13.57 kB). Data are available under the terms of the Creative Commons Zero “No rights reserved” data waiver (CC0 1.0 Public domain dedication).
