# Supplementary material for: Evaluation of serum complement C4 among COVID-19 patients in Khartoum state: a case control-based study
Source: F1000Res. 2023 Apr 21;12:432. [Version 1] doi: 10.12688/f1000research.132670.1 (PMC10390792; doi:10.12688/f1000research.132670.1)
Supplement: Peer review history

# Reviewer report: Reviewer response for version 1

**DOI:** 10.5256/f1000research.145606.r188619

**Published:** 2023-10-16

**Authors:** Andang Miatmoko

**Affiliations:** 1 Universitas Airlangga, Surabaya, East Java, Indonesia

This manuscript provides supporting data regarding the profiles of complement C in healthy subjects and COVID-19 patients with different severity of illnesses. Some information needs to be clarified or confirmed to improve the quality of the manuscript. 

1. Please give more details about the roles of complement C in the progression of infectious diseases so the manuscript's main idea can be clearly defined. In addition, the authors have cited research that shows the profile of complement c in COVID-19 (Ref.8). So, what is this study's new information or added value? Please define it clearly. 
2. In this study, the authors did not provide details of medication taken or consumed by the subjects. Please add the details since using different classes of antipyretics may affect the complement c levels.
3. Regarding mild and severe cases, the authors defined them based on ICU and non-ICU cases. However, ICU patients often have comorbidities, causing poor prognosis of the disease. This study focuses on complement C and the severity level of COVID-19, so it needs specific data regarding the severity of COVID-19, for example, Ct values or any other COVID-19 markers. 
4. Please describe when the blood sampling was conducted in the method section. In addition, the incubation period of infection may affect the levels of complement c, and it can be analyzed from the day patients suffered from the symptoms and the day they were admitted to the hospital. Please add the data about this. Or, could the author provide the references that the incubation period does not affect the levels of complement c.
5. Since the profiles of serum complement levels between control and case, and mild case and severe case had different results, which is affected by the immune response, please add the data regarding blood neutrophils so the analysis can be carried out well. 

Is the work clearly and accurately presented and does it cite the current literature?

Yes

If applicable, is the statistical analysis and its interpretation appropriate?

Yes

Are all the source data underlying the results available to ensure full reproducibility?

Yes

Is the study design appropriate and is the work technically sound?

Yes

Are the conclusions drawn adequately supported by the results?

Yes

Are sufficient details of methods and analysis provided to allow replication by others?

Yes

Reviewer Expertise:

Pharmaceutical Sciences

I confirm that I have read this submission and believe that I have an appropriate level of expertise to confirm that it is of an acceptable scientific standard, however I have significant reservations, as outlined above.

# Reviewer report: Reviewer response for version 1

**DOI:** 10.5256/f1000research.145606.r185092

**Published:** 2023-07-31

**Authors:** Marwa Hameed Alkhafaji

**Affiliations:** 1 University of Baghdad, Baghdad, Baghdad Governorate, Iraq

This manuscript includes a case-control study that attempted to correlate complement C4 levels with COVID-19 infection pathogenesis and outcome. The complement C4 level in the case group was lower than in the controls. The manuscript material could be viewed as additional support for the previous research highlighting the role of complement in the pathophysiology of COVID-19.

 Despite the fact that the manuscript is nicely written, and adds to the current literature, I have some comments and questions that should be addressed by the author:

- The introduction is excessively brief and lacks critical information about the correlation between complement and COVID-19 pathogenesis, with only one paragraph detailing this crucial part. As a result, authors should cite some recent papers to validate their findings.

- Section of the methodology:
  1. How and at what power was the sample size estimated?
  2. The optimal Cases to Control ratio should be 1:4, but the authors used 1:1 why?
  3. Why were patients with immunological dysfunction or malignant neoplasm excluded from the study? Inclusion and exclusion criteria section? It should be clearer or deleted.
- Results: This section is well-written, and the statistical analysis and interpretation are precise.
- In the discussion part, ''Prior clinical research has demonstrated that, whereas males and females share a comparable sickness rate, males are more likely to be hospitalized, experience a more severe illness progression, and die at a higher rate than females'' - is this comment a criticism or an interpretation?
- Conclusions: Well written and accurately reflects the author's findings.

Is the work clearly and accurately presented and does it cite the current literature?

Yes

If applicable, is the statistical analysis and its interpretation appropriate?

Yes

Are all the source data underlying the results available to ensure full reproducibility?

Yes

Is the study design appropriate and is the work technically sound?

Yes

Are the conclusions drawn adequately supported by the results?

Yes

Are sufficient details of methods and analysis provided to allow replication by others?

Yes

Reviewer Expertise:

Microbiology

I confirm that I have read this submission and believe that I have an appropriate level of expertise to confirm that it is of an acceptable scientific standard.

# Response

**Published:** 2023-08-01

**Authors:** Mohammed Yahya

**Affiliations:** Microbiology, Sudan University of Science and Technology, Khartoum, Khartoum, Sudan

Thanks you for your time to review our manuscript

 In the introduction part

 1. Regarding the introduction part we will make the required modifications which will appear in the second version of the manuscript.

 In the methodology part

 2. Regarding the sample size I would like to inform you that we used a total coverage sampling technique therefore we involved all COVID-19 patients in specific time manner and we use 1:1 case to control ratio because this is the least ratio that can be applied for sampling the case control study population and it's acceptable in many previous investigations.

 3. patients with immune dysfunction or malignant neoplasm were excluded be uses such disorders will affect the immune Status of cases and controls and consequently will affect on the complement level including C4 and according this will  lead to bias in the results therefore we excluded them from the cases and controls.

 5. in the discussion part

 Prior clinical research has demonstrated that, whereas males and females share a comparable sickness rate, males are more likely to be hospitalised, experience a more severe illness progression, and die at a higher rate than females. This section is interpretative part.

# Reviewer report: Reviewer response for version 1

**DOI:** 10.5256/f1000research.145606.r182340

**Published:** 2023-07-04

**Authors:** Adnan Mezher Maysam

**Affiliations:** 1 College of Pharmacy, Tikrit University, Tikrit, Saladin Governorate, Iraq

I think this article is an important addition to the literature, because the complement is a vital component of innate immunity where it forms the first line of defense against potentially harmful microbes, but its role in COVID-19 is still not clear. In this article, the results showed that case group had a higher level of complement C4 than the control group, which means that the complement plays an important role in the severity of disease, and the C4 complement should be considered as a diagnostic marker.

 I hope the authors intend to evaluate level of anti-pro inflammatory cytokine, such as IL-37 and IL-38, soon in severe and non-severe COVID-19 cases.

 I have carefully read this manuscript and below are my comments:

- Are the authors discussing the role of complement in pathogenesis of COVID-19 patients or in immune system? Clear this point.
- Indicate the aim of this paper more clearly and the conclusion also needs improvement.

Is the work clearly and accurately presented and does it cite the current literature?

Yes

If applicable, is the statistical analysis and its interpretation appropriate?

Yes

Are all the source data underlying the results available to ensure full reproducibility?

Yes

Is the study design appropriate and is the work technically sound?

Yes

Are the conclusions drawn adequately supported by the results?

Yes

Are sufficient details of methods and analysis provided to allow replication by others?

Yes

Reviewer Expertise:

Clinical immunology, COVID-19

I confirm that I have read this submission and believe that I have an appropriate level of expertise to confirm that it is of an acceptable scientific standard.

# Response

**Published:** 2023-07-04

**Authors:** Mohammed Yahya

**Affiliations:** Microbiology, Sudan University of Science and Technology, Khartoum, Khartoum, Sudan

Dear Dr. Adnan Mezher Maysam

 Thank you for your time to review our mansucript. 

 We carefully read your comments and this is our reply please track in a manner that comment followed by the authors response 

 Comment 1:

 I hope the authors intend to evaluate level of anti-pro inflammatory cytokine, such as IL-37 and IL-38, soon in severe and non-severe COVID-19 cases.

 Authors response:

 Thank you for your comment and we will consider it in our near future work to understand the role of these anti - pro inflammatory cytokine in the COVID-19 infection progression 

 Comment 2 

 Are the authors discussing the role of complement in pathogenesis of COVID-19 patients or in immune system? Clear this point

 Authors response:

 Actually this manuscript is discussing the role of complement C4 in the progression of COVID 19 infection and it was based on the evidence that complements are more likely to be consumed among severly ill COVID-19 patient due to development of complication like DIC  so the manuscript is more related to pathogenesis of COVID-19 based on immunological activity. 

 Comment 3

 Indicate the aim of this paper more clearly and the conclusion also needs improvement.

 Authors response:

 Thank you for your comment we will carefully consider it in the revised version of the manuscript 

 Again thank you for your time and efforts
